# Micro-shear bond strength of 3D printed hybrid ceramic with non-thermal plasma surface treatment: in-vitro study

**DOI:** 10.1038/s41598-026-43647-w

**Published:** 2026-04-02

**Authors:** Mostafa El-Shazly, Ghada Alkaranfilly, Mahmoud Osama El-Ghazawy, Bassem Emad, Aliaa Mahrous

**Affiliations:** 1 Fixed Prosthodontics department, Faculty of Dentistry, Fayoum University, Fayoum, Egypt; 2 Dental Biomaterial Department, Faculty of Dentistry, Fayoum University, Fayoum, Egypt; 3 Royal College of surgeons of Edinburgh, Edinburgh, UK; 4 Prosthodontics department, October university for modern science and arts ( MSA university), Giza, Egypt

**Keywords:** Low-temperature plasma, Air abrasion, Resin-dentin bonding, Materials science, Medical research

## Abstract

​ The optimal surface treatment for developing a durable bond of dual-cure self-adhesive resin cement and 3D printed hybrid ceramic has not yet been established. To evaluate the effect of Non-Thermal Atmospheric Plasma (NTAP), sandblasting (SB), and a combination of both techniques on Micro-Shear Bond Strength (µSBS) of dual-cure self-adhesive resin cement bonded to 3D printed hybrid ceramic after thermocycling, and assess the failure mode using SEM. A total of 75 resin tags of dual-cure self-adhesive resin cement (TheraCem) were cemented to fifteen discs of 3D Printed Hybrid Ceramic (Saremc- Print Crowntec) (five resin tags/ disc). Specimens were allocated into five groups according to discs’ surface treatment (n = 15); PL group: specimens were treated with NTAP, S50 group: specimens were treated with 50 μm AL2O3 SB, S110 group: treated with 110 μm AL2O3 SB, SP50 group: treated with 50 μm AL2O3 SB followed by NTAP, and SP110 group: treated with 110 μm AL2O3 SB followed by NTAP. Resin cylinders, 0.8 mm in diameter and 1 mm height, were cemented to discs. The µSBS test was performed using a universal testing machine after thermocycling. SEM was used to analyze the failure mode. Data were analyzed using Welch one-way ANOVA followed by the Games-Howell post hc test. A significant difference between groups (P = 0.026). The highest value was measured in SP50 (2.70 ± 0.49) (MPa), followed by SP110 (2.37±0.38) (MPa), then PL (2.35 ± 0.57) (MPa), and S110 (2.18 ± 0.31) (MPa), while the lowest value was found in S50 (2.12 ± 0.75) (MPa). Post hoc pairwise comparisons showed SP50 to have significantly higher values than S110 and S50 (P < 0.05). NTAP, particularly when combined with 50 μm sandblasting, improved µSBS to 3D-printed hybrid ceramics under the conditions of this in-vitro study. Clinical validation is recommended.

**Supplementary Information:**

The online version contains supplementary material available at 10.1038/s41598-026-43647-w.

## Introduction

The evolution in development of digital scanners, milling machines, and 3D printers made digital work flow a better and stronger alternative than conventional manufacturing techniques. (CAD/CAM technology encompasses both subtractive (milling) and additive (3D printing) manufacturing techniques. While milling is widely established, it is often hindered by high costs, substantial spatial requirements, and the need for frequent maintenance. Furthermore, the milling process can be labor-intensive, necessitating significant operator intervention which may increase the margin for human error^1,2^.

Another limitation of subtractive dental milling lies in its inability to fabricate highly complex geometries; this limitation stems from the physical dimensions of the milling burs and the limited freedom provided by the machine’s axes. Also, many researchers considered the milling technology as non-environmentally friendly technique because of the produced waste material, heat, noise, and dust^1^.

Compared to milling, 3D printing offers superior geometric freedom, enabling the fabrication of complex designs and minute features that exceed the capabilities of subtractive tools. Additionally, the process minimizes material waste and facilitates the concurrent production of multiple units, significantly improving clinical and laboratory workflows. 3D printing significantly lessen the thermal accumulation and micro-fracture initiation typically associated with the high-speed friction of subtractive milling burs. By eliminating these mechanical stressors, 3D printing preserves the structural integrity of the restorative material. Furthermore, current literature suggests that 3D-printed crowns demonstrate superior marginal and internal adaptation compared to milled crowns, offering a more precise fit for complex dental prosthetics^1,3^.

Ceramics and composite resins represent the primary material classes utilized for definitive dental restorations. In terms of clinical handling, composite resins demonstrate superior repairability, and ease of finishing. Furthermore, they exhibit lower abrasion toward antagonist dentition compared to more rigid ceramic structures. However, composite materials are inherently limited by inferior flexural strength, surface hardness, and fracture toughness. Additionally, they are prone to reduced optical stability and rapid surface degradation over time^4,5^.Ceramics are stronger, harder, and more biocompatible than composite with less plaque accumulation, no residual monomers, which provide better biocompatibility with periodontal tissue^6^. But Ceramics are hard to repair, and fragile. Literature indicates that while ceramics offer superior aesthetic outcomes, their clinical longevity is frequently compromised by two primary factors: the accelerated attrition of antagonist dentition and a predisposition to catastrophic failure resulting from subcritical crack propagation^6^.

Hybrid ceramics represent an innovative class of materials that merge the favorable properties of ceramics and composites^7^. Their structure mitigates brittleness and high surface hardness, thereby reducing antagonist wear, facilitating repair, and enabling efficient milling, which collectively may enhance clinical performance^8^.

There are many types of hybrid ceramic CAD/CAM blocks: Vita Enamic (VE), a polymer-infiltrated ceramic network; Lava Ultimate (LU), a resin nanoceramic; and Cerasmart, a hybrid ceramic with nanofiller particles^7^.

Multiple 3D printable resin-based hybrid materials for permanent restorations are now available on the US market, including VarseoSmile Crown Plus, Permanent Crown (Formlabs), Flexcera^™^Smile Ultra+, and Saremco Print CROWNTEC^9,10^.

Durable bonding between the restoration and resin cement is critical for long-term clinical success^11,12^. However, hybrid ceramic restorations remain susceptible to debonding and fracture, which can compromise their performance^13,14^. In order to enhance bonding of 3D printed hybrid ceramic material to resin cement, the manufacturer recommends sandblasting (SB) using 110 μm particles, but sandblasting can lead to damage of restoration surface, defects, and cracks^15^. Therefore, the mechanical characteristics of ceramics can be compromised^16,17^. It is advisable to carry out sandblasting according to adequate parameters in relation to pressure, distance from the source, and particle size^18,19^.

Non-thermal atmospheric plasma (NTAP) has emerged as a promising surface treatment in dentistry. NTAP is an ionized gas containing excited atoms, molecules, and radicals that can be generated at atmospheric pressure and near room temperature^20,21^. NTAP modifies surfaces at a molecular level, enhancing surface energy, removing contaminants, and improving wettability without altering bulk material properties^22–24^. Plasma has different forms, including natural and artificial types. It can also be classified according to its parameters: at the atmospheric pressure level, there is a thermal type and cold plasma (non-thermal) with a gas temperature close to room temperature^25,26^.

The introduction of Non-Thermal Atmospheric Plasma (NTAP), which can generate plasma jets at room temperature under atmospheric conditions, has enabled extending the use of plasma to the field of dentistry, with reported applications such as tooth whitening, modifying implant surfaces to promote osteointegration and soft tissue healing, and increasing ceramic/adhesive interfacial bonding^24,27,28^.

This study evaluated the effect of NTAP surface treatment and SB surface treatment on µSBS of dual-cure self-adhesive resin cement bonded to 3D printed hybrid ceramic discs after thermocycling, as well as the mode of failure using scanning SEM. The null hypothesis was that there was no significant difference in the µSBS of dual-cure self-adhesive resin cement bonded to 3D printed hybrid ceramic discs with different surface treatments. While previous studies have explored NTAP on milled hybrid ceramics, its effect on 3D-printed hybrid ceramic materials which differ in composition, polymerization behavior, and surface reactivity, remains underexplored. This study specifically investigates the efficacy of NTAP on a representative 3D-printed hybrid ceramic (Saremco Print CROWNTEC), providing new data relevant to the growing adoption of additive manufacturing in permanent restorations.

## Materials and methods

A sample size calculation (*n*= 15) was performed using G*Power version 3.1.9.4. with a desirable al power of 95% and a 95% confidence interval (α = 0.05)^29^.

Fifteen discs with 10 mm diameter and 1.5 mm thickness were designed using 3D software (Meshmixer; Autodesk), and printed using Hybrid Ceramic (Saremco, Dental AG) by digital light processing technology-based 3D printer (MAX UV; ASIGA.). Discs were oriented at 0 degrees (parallel to the build platform) with a 50 μm slice thickness and a working temperature of 35 °C/95°F according to the manufacturer’s instructions.

Discs were removed from the platform, and the supports were removed with a carbide rotary bur (FG SL-701, ISO 012, SSWHITE). Discs were cleaned with a brush soaked in alcohol (Isopropyl alcohol, ≥ 99%) until all resin remains were completely removed, and thoroughly dried with compressed air. Polymerization was completed in a UV-light box (LC- 3DPrint Box; NextDent.) for 30 min, and placed in boiling water (100 °C) for 2 min according, to the manufacturer’s instructions to complete the post-curing process^30^.

Finishing of all discs were completed with a #240, #320, and #600 silicon carbide grinding paper (Struers, Cleveland, OH) for 10 s using grading machine (EcoMet 30 Grinder- Polisher; Buehler Co) under water cooling with light contact pressure according to manufacturer’s recommendations. Discs were designed, fabricated, processed, finished, and polished by a single operator.

Discs were randomly allocated to 5 groups (*n* = 3 per group) using a computer-generated simple randomization sequence performed by a blinded operator.

### Grouping

(PL) group: surface was treated with a hand-held NTAP (Piezobrush^®^ PZ2, Relyon plasma GMBH) using ambient air as the working gas, which generated a plume of plasma jet at atmospheric pressure. The nozzle of the NTAP device was fixed at a distance of 10 mm from the specimens for 80 s at a maximum power consumption of 30 W and a temperature of ~ 50 °C. The 80‑second exposure was selected based on pilot testing and prior studies indicating that this duration achieves consistent surface activation without thermal damage.

(S50) group: surface was sandblasted with a standard nozzle with an internal diameter of 1.2 mm using 50 μm AL2O3 particles (Renfert Basic Blassic, Renfert GmbH) at 0.2 MPa pressure for 10 s from a distance of 10 mm perpendicular to the disc’s surface. (S110) group: same as (S50) group, but the AL2O3 particles size was 110 μm.

(SP50) group: surface was sandblasted using 50 μm AL2O3 particles at 0.2 MPa pressure for 10 s from a distance of 10 mm perpendicular to the disc’s surface and followed by NTAP following the same protocol as (PL) group. (SP110) group: same as (SP50) group, but AL2O3 particles size was 110 μm.

A slice of microbore Tygon tubing (TYG-030 Tygon tubing, Saint Gobain Performance Plastic) with an internal diameter of 0.8 mm and a height of 1 mm was positioned over discs and carefully filled with dual-cure self-adhesive resin cement (TheraCem, Bisco). Initial curing for 2 s was done and then oxygen barrier gel was applied and the resin cement was light-cured using an LED curing light (Elipar™ Deep Cure-S LED Curing Light, 3 M-ESPE.) for 20 s according to manufacture recommendation

Five resin cylinders were built on each disc, totaling 75 cylinders (15 cylinders per group) discs were stored in distilled water at 37 °C for 24 h in an incubator (Sanyo Electric Company Ltd) to completely polymerize prior to removal of Tygon tubing. Then Specimens were thermocycled (5000 cycles; between 5 °C and 55 °C, dwell time of 30 s) using a Jencons Julabo FT200 circulating water bath (Jencons Scientific Ltd)^31^.

Discs were mounted in epoxy resin moulds, and labelled with a specific number for each group. So, the type of surface treatment was blinded to the examiner.

### Samples testing

**Fig. 1:**
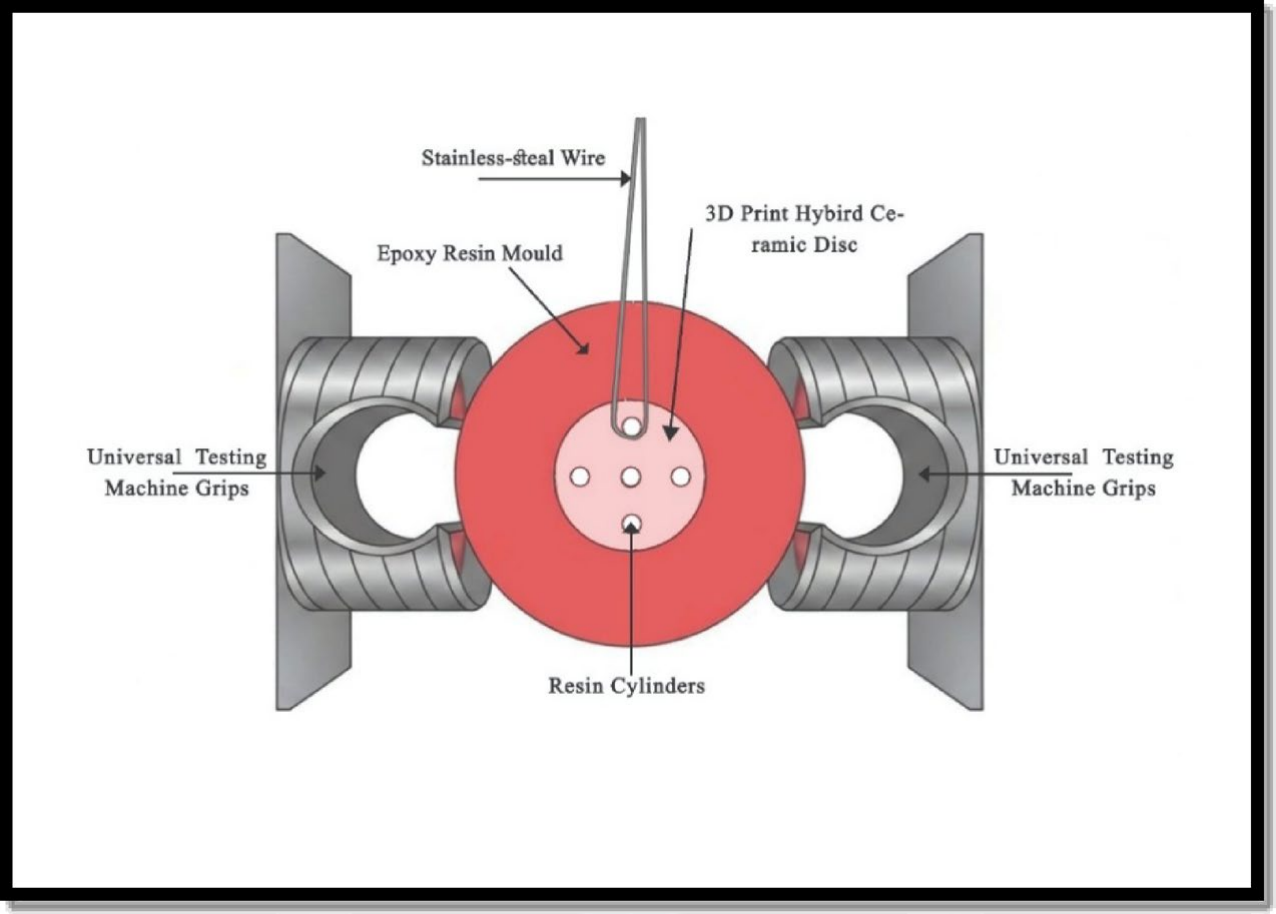
Diagram of horizontally secured disc in the universal testing machine.

µSBS test was conducted using a universal testing machine (Model 3345; Instron Industrial Products) with a stroke length of 50 mm & a peak force of 250 N, and with a resolution of 0.5 microns. A 0.2 mm in diameter stainless-steel wire was looped around the base of the specimens to contact half its circumference and was held on the other end against the fixed metal turret on the jig (Fig. 1). The stainless-steel wire was positioned as close as possible to the adhesive interface to apply shear force parallel to the bonded interface, ensuring a predominantly shear loading configuration. A shear load was applied at a crosshead speed of 1 mm/minute until failure^32,33^. µSBS values were calculated in MPa by dividing the load at failure (N) by the surface area of each cylinder (mm²)^34^. As follows:

$$\mu SBS\left( {MPa} \right) = \frac{{load~of~failure(N)}}{{surface~area~for~each~cylinder(mm^{2} )}}$$

Specimens were examined under a magnifying lens (Glass 10X Handheld Magnifier 75 MM.) to de^35^termine the failure pattern. Failure mode was categorized into 3 main groups and each group was subdivided into subgroups as follows: (Table 1)

**Table 1:** Failure mode categorization.

Types	Description
Adhesive failure (A)	Interfacial debonding across hybrid ceramic-resin cement adhesive interface
Cohesive failure (C)	C1. Debonding within hybrid ceramic. C2. Debonding within resin cement.
Mixed failure (M)	M1. Adhesive interface and cohesive hybrid ceramic. M2. Adhesive interface, cohesive hybrid ceramic, and cohesive resin cement. M3. Adhesive interface and cohesive resin cement.

The debonded surfaces were analyzed for all morphological and ultra-structural changes using a Scanning Electron Microscope (SEM) (VEGA3, Tescan.) with an accelerating voltage of 30 kV, ×14 magnification (up to 1,000,000), and a secondary electron detector for the gun.

### Statistics

The micro-shear bond strength data were assessed for normality and homogeneity of variances using Shapiro-Wilk’s test. Data were positively skewed and transformed using Cox transformation (square root) to achieve normality. The variance homogeneity assumption was checked using Levene’s test and was found to be violated. Intergroup comparisons were analyzed using Welch one-way ANOVA followed by the Games-Howell post hoc test. The significance level was set at *P* < 0.05. Statistical analysis was performed with R statistical analysis software version 4.3.3 for Windows.

## Results

Descriptive statistics for micro-shear bond strength (MPa) values are presented in (Table 2).

**Table 2:** Descriptive statistics.

Group	Mean ± SD	95% Confidence interval	Min.	Max.
PL	6.40 ± 3.01	4.87–7.92	1.02	13.97
S50	5.50 ± 3.96	3.50–7.51	1.00	13.47
S110	5.28 ± 1.57	4.48–6.07	3.25	8.48
SP50	8.35 ± 3.12	6.77–9.93	3.40	14.35
SP110	6.29 ± 2.06	5.25–7.33	3.00	9.83

According to the results, there was a significant difference between different groups (*P*=0.026). The highest mean µSBS value was recorded for the SP50 group (2.70 ± 0.49 MPa), followed by the SP110 (2.37 ± 0.38 MPa) and PL (2.35 ± 0.57 MPa) groups. The S110 (2.18 ± 0.31 MPa) and S50 (2.12 ± 0.75 MPa) groups showed lower mean µSBS values. Post hoc pairwise comparisons using the Games–Howell test demonstrated that the SP50 group exhibited significantly higher µSBS values than the S110 and S50 groups (*P* < 0.05). No statistically significant differences were detected among the remaining group comparisons (Table 3).

**Table 3:** Intergroup comparisons, mean and standard deviation values of micro-shear bond strength (MPa) (*transformed*).

Groups	Mean ± SD
PL	2.35 ± 0.57^AB^
S50	2.12 ± 0.75^B^
S110	2.18 ± 0.31^B^
SP50	2.70 ± 0.49^A^
SP110	2.37 ± 0.38^AB^
p-value = 0.026^*^	

^*^ significant (*p* < 0.05). Values with different superscripts within the same horizontal row are significantly different and values with same superscripts within the same horizontal raw are insignificantly different.

**Fig. 2:**
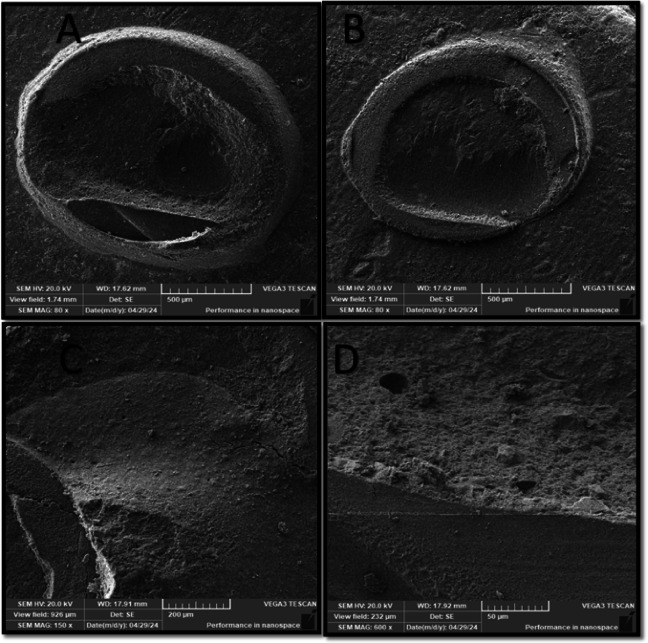
SEM photomicrograph of PL group showing: (**A**) Magnifications (80x) showed cohesive failure with crack lines within the resin cement. (**B**) Mixed failure (M2) with cohesive ceramic part extend for 50% of the surface. (**C**) Higher magnifications (150x) showed crack lines of the surface with no detachment of resin cement. (**D**) Magnifications (600x) showed a nearly homogenous layer of resin cement with some dragged parts of the surface.

**Fig. 3:**
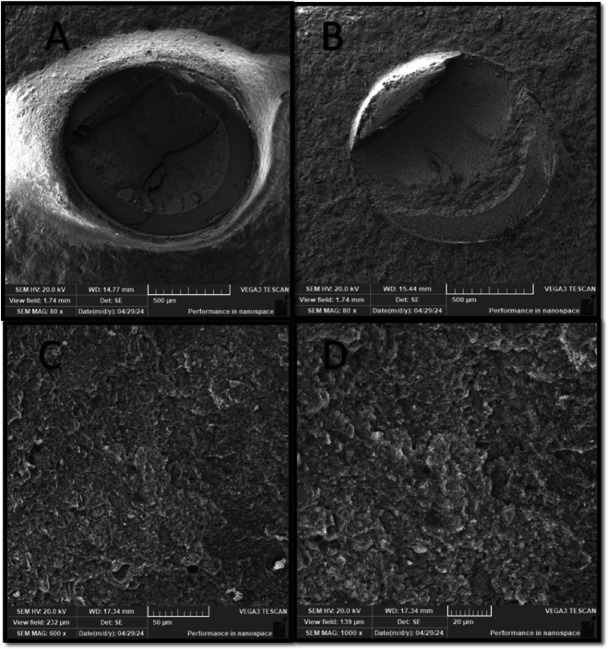
SEM photomicrograph of S110 group showing: (**A**) Magnification (80x) showed cohesive failure mode within the hybrid ceramic with clear depressed areas of the surface. (**B**) Mixed failure mode (M1) presented as large area of cohesive failure within the hybrid ceramic. (**C**) Higher magnifications (150x) showed failure of the hybrid ceramic as a striated pattern. (**D**) Magnifications (600x) showed thick resin layer with many finger-like projections and deeper striations.

**Fig. 4:**
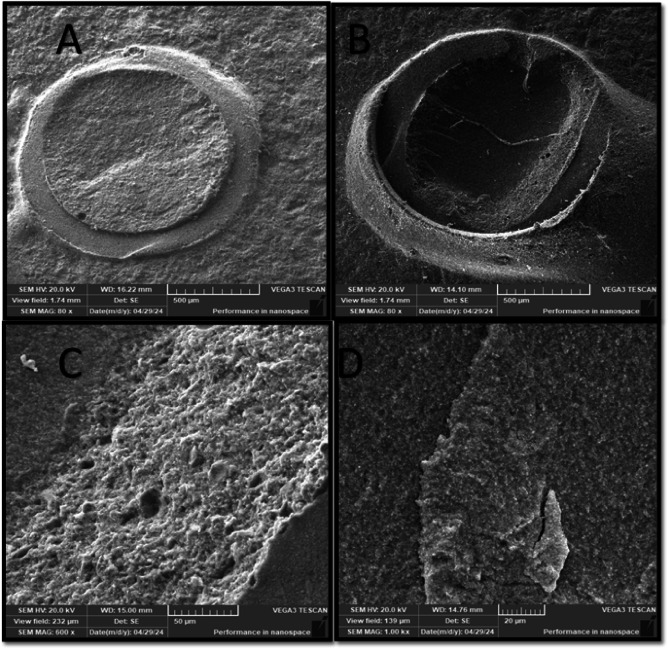
SEM photomicrograph of SP50 group showing: (A), Magnification (80x) showed cohesive failure mode within resin cement, which looked like having different layer thickness. (B) Mixed failure mode (M3), where failure was about 50% adhesive at the interface. (C) Higher magnifications (600x magnification) showed that the resin looked to be interdigitated with the hybrid ceramics, which was impossible to differentiate. (D) Magnifications (1000x) showed a thick layer of resin cement covering the surface..

**Fig. 5:**
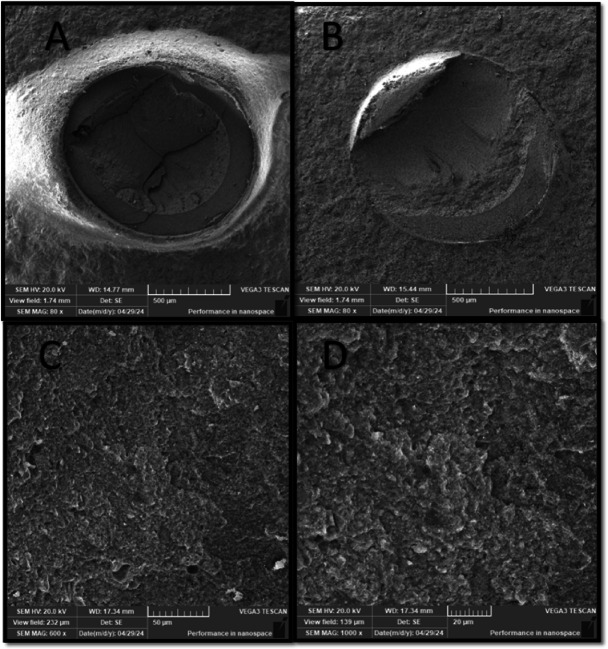
SEM photomicrograph of SP110 group showing: (A) Magnification (80x) showed cohesive failure within the hybrid ceramic with fractured lines. (B) Mixed failure mode of type (M2) with hybrid ceramic appeared as if it was dragged from the resin cement. (C), Higher magnifications (600x magnification) showed irregularly distributed resin on the surface. (D) Magnifications (1000x) showed separate clusters of thick layers of the resin cement.

Failure mode distribution is summarized in Table 4. Adhesive, cohesive, and mixed failure patterns were observed across all experimental groups. The SP50 group demonstrated cohesive and mixed failure modes, whereas adhesive failure was not observed in this group. Representative scanning electron microscope images of the failure modes are presented in (Figs. 2, 3, 5 and 4).

**Table 4:** Failure mode categories.

failure groups	Adhesive	Cohesive		Mixed			Total
	A	C1	C2	M1	M2	M3	
PL	3	0	5	2	2	3	15
S50	1	3	1	3	5	2	15
S110	2	5	0	4	4	0	15
SP50	0	2	6	1	2	4	15
SP110	1	2	0	4	3	5	15

## Discussion

Digitalization has become increasingly prevalent in dentistry and is now an integral part of modern practice. 3D printing technology is still relatively new, yet there numerous current applications demonstrate its growing popularity^2^.

Newly developed 3D printed hybrid ceramic is a revolutionary design for the dental field. This innovative material combines the benefits of hybrid ceramic restorations with the advantages of 3D printing technology. 3D printed hybrid ceramic Saremco Print Crowntec features allow for excellent aesthetics, colour stability, resistance to wear, high flexural strength, intraoral repair capability, and durability, making it being used in a wide range of dental applications, including crowns, bridges, inlays, onlays, and veneers^10,13,36^.

The surface treatment of hybrid ceramic is a challenge in creating a strong and durable bond. Surface treatment protocols are essential to enhance topographical roughness and elevate surface energy. These changes enhance hydrophilic interface, which is critical for ensuring optimal wettability and micromechanical interlocking with adhesive agents^11^. For hybrid ceramic restorations, sandblasting surface treatment is recommended to enhance bonding between the restoration and resin cement. But It has been documented that sandblasting serves as an initiator for surface defects and cracking, which compromise the mechanical properties of ceramic materials^16^.

The most appropriate test for the present study was the µSBS test. This can be explained as it’s well-known that tensile bond strength tests were greatly affected by the strength of the ceramic itself and not by the true adhesive bond with the resin cement, also µSBS test provides more precise and reproducible results, as it is less affected by surface roughness and other factors that can influence the bonding interface^32,33^.

According to the results, the Alternative Hypothesis stated that “There was a significant difference in the micro-shear bond strength of dual-cure self-adhesive resin cement bonded to 3D printed hybrid ceramic discs with different surface treatments” was accepted.

The highest bond strength was measured in NTAP surface treatment groups (SP50, SP110, and PL). These µSBS values (ranging from 2.12 to 2.70 MPa) fall within the range reported for resin-ceramic bonding in the literature. While no universal threshold for clinical success exists, bond strengths above 1.8–2.0.8.0 MPa are generally considered acceptable for adhesive cementation of indirect restorations.

These findings corroborate the observations of Xiaoming Zhu et al.^22^, and Mahmoud El-Said et al.^19^, who reported that NTAP significantly enhances the bond strength between resin cement and hybrid ceramics. This surface modification occurs without altering the bulk properties of the substrates, thereby reducing the risk of inducing micro-fissures or structural cracks. NTAP modifies material surfaces primarily through physicochemical activation rather than mechanical alteration. Plasma treatment increases surface energy and wettability by removing organic contaminants and introducing reactive functional groups, thereby enhancing interaction between the hybrid ceramic surface and resin cement^26^.

On the other side, findings by Castro EF et al.^35^ represented a negative result that no significant benefit was found in hybrid ceramics’ bonding, using NTAP alone or combined with another surface treatment. These discrepancies may be attributed to variations in the NTAP devices and their specific operating parameters. For instance, their protocol utilized a plasma application of only 30 s, whereas the present study employed an 80-second duration. Furthermore, the hybrid ceramic CAD blocks used in their research featured distinct filler particle compositions compared to the 3D-printed hybrid ceramic utilized in this study, likely resulting in a differential response to NTAP treatment.

3D-printed materials are characterized by higher organic content and the presence of residual monomers after printing and curing. Therefore, the influence of chemical surface treatments, rather than mechanical sandblasting alone, is more pronounced in enhancing the bond strength of 3D-printed hybrid ceramics compared to milled materials^28^.

Accordingly, the present findings are most directly applicable to Saremco Print CROWNTEC, and extrapolation to other 3D-printed hybrid ceramics should be made with caution, as differences in resin matrix composition, filler content, and degree of conversion may influence surface reactivity and bonding behavior.

Zhen Mao et al.^12^reported that 3D-printable hybrid ceramic exhibit a distinctive response to surface treatments compared to millable ones. Specifically, for millable hybrid ceramics, the highest bond strength for VE was observed through sandblasting, Conversely, significant enhancements via silanization were exclusively observed in the 3D-printed hybrid surfaces. This divergence in adhesive behavior may be attributed to variations in the inorganic filler-to-matrix ratio and the resulting surface chemistry^14^. Beyond the effects of silanization, the chemical affinity of the 3D-printed substrate may be further enhanced by the 10-MDP functional monomers present in TheraCem resin cement. This phosphate monomer can undergo radical polymerization with available methacrylate groups on the hybrid ceramic surface, effectively cross-linking with the resin matrix to form a cohesive polymer network^37,38^.

The superior bond strength achieved in SP50 was attributed to the combination of NTAP and 50 μm AL2O3 sandblasting. These findings were in the same way of Mahrous et al.^24^, and Ahn JJ et al.^26^, who reported a greater bond strength of ceramics after NTAP treatment combined with SB. It was stated that SB can produce a roughened surface, providing more opportunities for NTAP reaction and a larger bonding area, without weakening the ceramic structure.

In contrast to the larger 110 μm AL2O3 particles used in the SP110 group, the smaller 50 μm AL2O3 particles used in the SP50 group have been shown to create a surface roughness that was more conducive to bonding with resin cement. This is because the small particles have more surface irregularities per unit area, and total bonding surface area. And, the larger 110 μm particles used in the SP110 group can damage the polymeric composition of the hybrid ceramic, leading to the removal of fillers and uneven infiltration of the cement. This explain why the bond strength observed in the SP50 group was significantly higher than in the SP110 group^19,34^.

These results align with the observations reported by Mahmoud El-Said et al.^19^, who demonstrated that using 50 μm Al2O3 surface SB resulted in significantly higher hybrid ceramic/cement bond strength compared to 110 μm Al2O3. The authors noted that excessively aggressive SB with 110 μm Al2O3 particles leads to the creation of microcracks on the ceramic surface, potentially causing premature failures.

The most striking finding was the predominance of cohesive failure within the resin cement in the SP50 group, whereas the SP110 group showed a higher proportion of cohesive failure within the hybrid ceramic. These observations were consistent with the SEM findings (Figs. 4 and 5), which demonstrated close interfacial adaptation between the resin cement and the hybrid ceramic surface.

In contrast, the PL group, which did not undergo sandblasting, displayed a higher proportion of adhesive and cohesive cement failures. SEM examination (Fig. 2) revealed the presence of crack lines within the resin cement without complete detachment from the hybrid ceramic surface, along with limited areas of resin displacement.

On the other hand, the S50 and S110 groups exhibited lower µSBS values and a higher proportion of ceramic cohesive failure, with no statistically significant difference between them.

These results are consistent with Xiaoming Zhu et al.^22^, who found that regarding the bonding of hybrid ceramic to resin cement, plasma treatment proved more effective than traditional sandblasting. And this also was supported by Elias et al.^21^, and Barquete et al^23^.

Finally, NTAP technique have introduced an effectiveness in 3D printed hybrid ceramic surface treatment, improving bond strength over SB, producing a more uniform and predictable surface treatment and bonding between 3D printed hybrid ceramic and resin cement.

This study has several limitations that should be considered. Micro-shear bond strength testing, while informative, does not fully replicate the complex functional loading and wet oral environment encountered clinically. The findings are based on a single 3D-printed hybrid ceramic material, one brand of dual-cure resin cement, and one NTAP device under fixed treatment parameters; thus, they may not be a general rule for other materials or protocols. Only two sandblasting particle sizes were tested, and other pressure, distance, or particle-type variations could yield different outcomes. Although surface roughness and contact angle measurements were not performed, they could provide complementary insight into the physicochemical effects of NTAP and sandblasting and are recommended for future studies.

Finally, although thermocycling provides an accelerated aging model, it cannot simulate all intraoral degradation factors, such as fatigue loading or long-term water sorption. Therefore, the results should be interpreted within the constraints of this in-vitro design, and clinical validation is warranted.

## Conclusion

Within the limitations of this in-vitro study, non-thermal atmospheric plasma treatment improved the micro-shear bond strength of 3D-printed hybrid ceramic to dual-cure self-adhesive resin cement, particularly when combined with 50-µm sandblasting. These findings are specific to the materials, surface treatments, and testing conditions employed. Further clinical studies are required to validate the applicability of this surface treatment protocol under intraoral conditions.

## Clinical significance

Non-thermal atmospheric plasma treatment, alone or in combination with sandblasting, shows potential as a surface treatment protocol for bonding 3D-printed hybrid ceramic restorations; however, clinical confirmation is required.

## Supplementary Information

Below is the link to the electronic supplementary material.

Supplementary Material 1

Supplementary Material 2

Supplementary Material 3

Supplementary Material 4

Supplementary Material 5

Supplementary Material 6

Supplementary Material 7

Supplementary Material 8

Supplementary Material 9

Supplementary Material 10

## Data Availability

The datasets used and/or analysed during the current study are available from the corresponding author on reasonable request.

## Abbreviations

µ: Mean
SD: Standard deviation
CI: Confidence level
Sig: Significance level
