# Supplementary material for: Micro-shear bond strength of 3D printed hybrid ceramic with non-thermal plasma surface treatment: in-vitro study
Source: Sci Rep. 2026 Apr 2;16:11237. doi: 10.1038/s41598-026-43647-w (PMC13046835; doi:10.1038/s41598-026-43647-w)

Figure 3 SEM photomicrograph of SP50 group showing: A, Magnification (80x) showed cohesive failure mode within resin cement which looked like having different layer thickness. B, Mixed failure mode (M3) where failure was about 50% adhesive at the interface. C, Higher magnifications (600x magnification) showed that resin looked to be interdigitated with the hybrid ceramics which was impossible to differentiate. D, Magnifications (1000x) showed a thick layer of resin cement covering the surface.

A
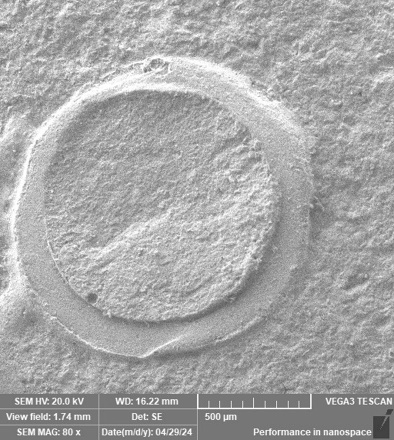
 B
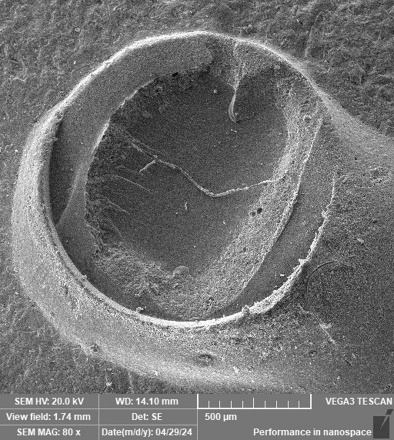
 C
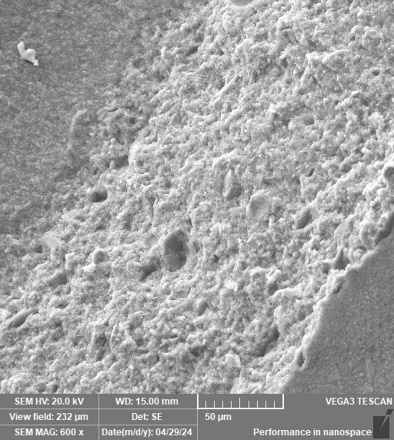


D
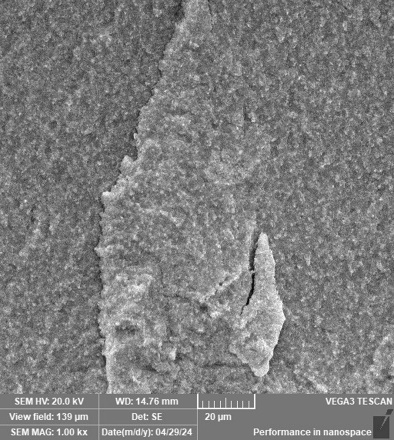

Supplement: Supplementary file 1 — Supplementary Material 1 [file 41598_2026_43647_MOESM1_ESM.docx]
