# Supplementary material for: Micro-shear bond strength of 3D printed hybrid ceramic with non-thermal plasma surface treatment: in-vitro study
Source: Sci Rep. 2026 Apr 2;16:11237. doi: 10.1038/s41598-026-43647-w (PMC13046835; doi:10.1038/s41598-026-43647-w)

Figure 4. SEM photomicrograph of SP110 group showing: A, Magnification (80x) showed cohesive failure within hybrid ceramic with fractured lines. B, Mixed failure mode of type (M2) with hybrid ceramic appeared as if it was dragged from the resin cement. C, Higher magnifications (600x magnification) showed irregularly distributed resin on the surface. D, Magnifications (1000x) showed separate clusters of thick layers of the resin cement.

A
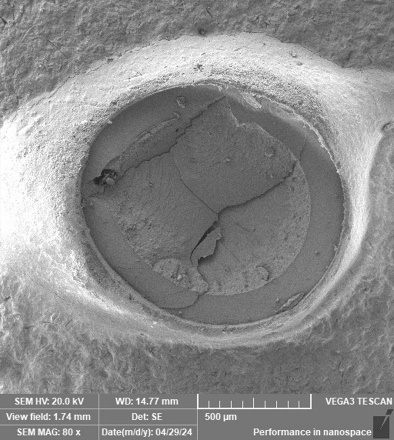
 B
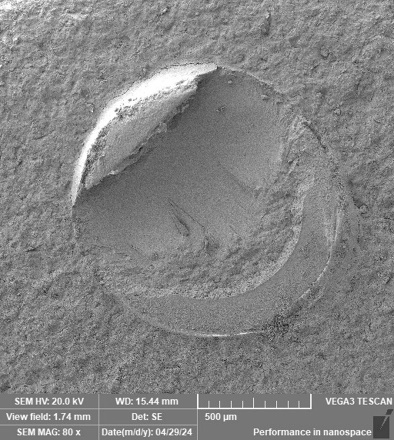
 C
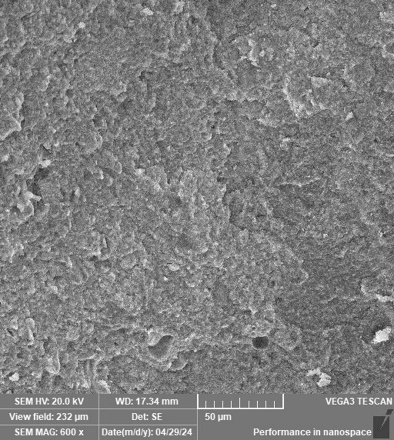


D
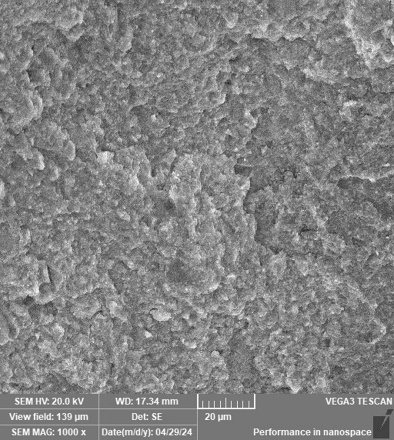

Supplement: Supplementary file 2 — Supplementary Material 2 [file 41598_2026_43647_MOESM2_ESM.docx]
