# Supplementary material for: Micro-shear bond strength of 3D printed hybrid ceramic with non-thermal plasma surface treatment: in-vitro study
Source: Sci Rep. 2026 Apr 2;16:11237. doi: 10.1038/s41598-026-43647-w (PMC13046835; doi:10.1038/s41598-026-43647-w)

Figure 2. SEM photomicrograph of S110 group showing: A, Magnification (80x) showed cohesive failure mode within hybrid ceramic with clear depressed areas of the surface. B, Mixed failure mode (M1) presented as large area of cohesive failure within hybrid ceramic. C, Higher magnifications (150x) showed failure of hybrid ceramic as a striated pattern. D, Magnifications (600x) showed thick resin layer with many finger-like projections and deeper striations.

A
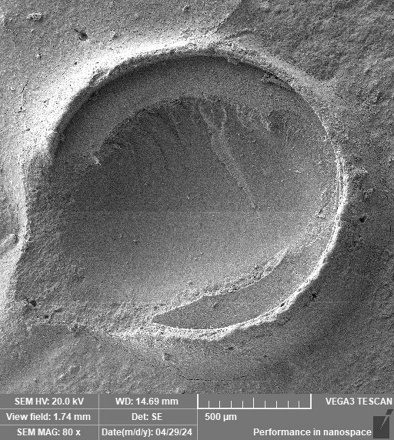
 B
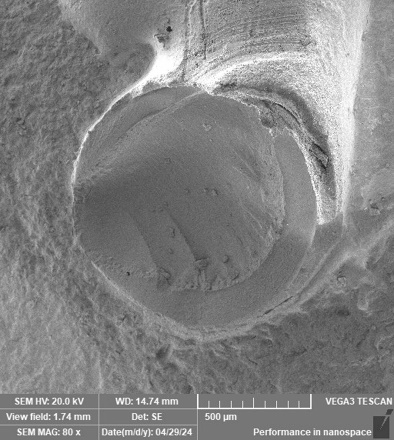
 C
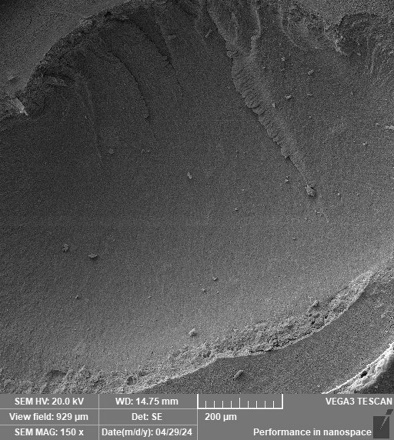


D
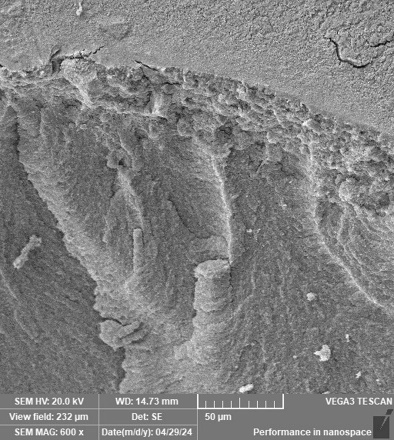

Supplement: Supplementary file 3 — Supplementary Material 3 [file 41598_2026_43647_MOESM3_ESM.docx]
