# Supplementary material for: Micro-shear bond strength of 3D printed hybrid ceramic with non-thermal plasma surface treatment: in-vitro study
Source: Sci Rep. 2026 Apr 2;16:11237. doi: 10.1038/s41598-026-43647-w (PMC13046835; doi:10.1038/s41598-026-43647-w)

Figure 1 SEM photomicrograph of PL group showing: A, Magnifications (80x) showed cohesive failure with crack lines within the resin cement. B, Mixed failure (M2) with cohesive ceramic part extend for 50% of the surface. C, Higher magnifications (150x) showed crack lines of the surface with no detachment of resin cement. D, Magnifications (600x) showed nearly homogenous layer of resin cement with some dragged parts of the surface.

A
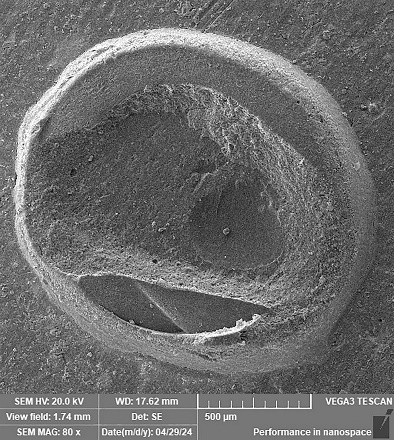
 B
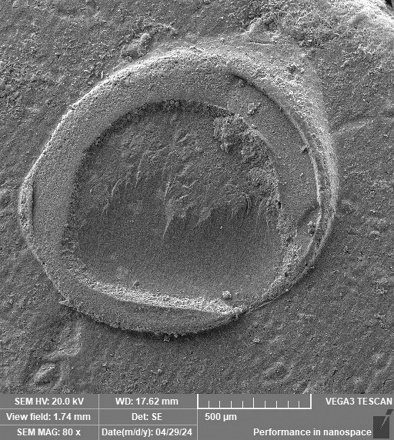
 C
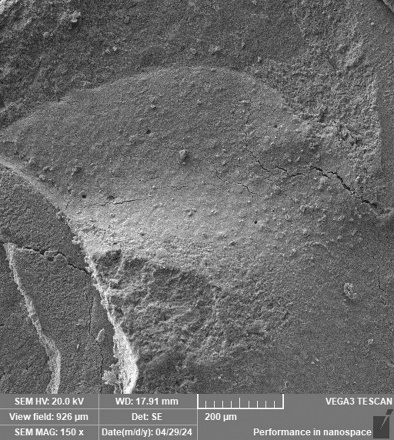


D
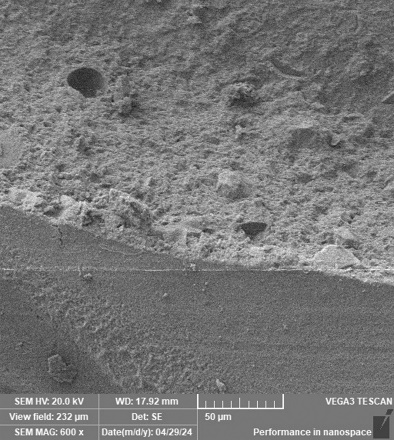

Supplement: Supplementary file 4 — Supplementary Material 4 [file 41598_2026_43647_MOESM4_ESM.docx]
