# Supplementary material for: Micro-shear bond strength of 3D printed hybrid ceramic with non-thermal plasma surface treatment: in-vitro study
Source: Sci Rep. 2026 Apr 2;16:11237. doi: 10.1038/s41598-026-43647-w (PMC13046835; doi:10.1038/s41598-026-43647-w)
Supplement: Supplementary file 5 — Supplementary Material 5 [file 41598_2026_43647_MOESM5_ESM.docx]

**Table 2:** Descriptive statistics.

| Group | Mean ± SD | 95% Confidence interval | Min. | Max. |
| --- | --- | --- | --- | --- |
| PL | 6.40±3.01 | 4.87-7.92 | 1.02 | 13.97 |
| S50 | 5.50±3.96 | 3.50-7.51 | 1.00 | 13.47 |
| S110 | 5.28±1.57 | 4.48-6.07 | 3.25 | 8.48 |
| SP50 | 8.35±3.12 | 6.77-9.93 | 3.40 | 14.35 |
| SP110 | 6.29±2.06 | 5.25-7.33 | 3.00 | 9.83 |
