# Supplementary material for: Micro-shear bond strength of 3D printed hybrid ceramic with non-thermal plasma surface treatment: in-vitro study
Source: Sci Rep. 2026 Apr 2;16:11237. doi: 10.1038/s41598-026-43647-w (PMC13046835; doi:10.1038/s41598-026-43647-w)
Supplement: Supplementary file 6 — Supplementary Material 6 [file 41598_2026_43647_MOESM6_ESM.docx]

**Table 4:** Failure mode categories

| failure  groups | Adhesive | Cohesive | | Mixed | | | Total |
| --- | --- | --- | --- | --- | --- | --- | --- |
|  | A | C1 | C2 | M1 | M2 | M3 |  |
| PL | 3 | 0 | 5 | 2 | 2 | 3 | 15 |
| S50 | 1 | 3 | 1 | 3 | 5 | 2 | 15 |
| S110 | 2 | 5 | 0 | 4 | 4 | 0 | 15 |
| SP50 | 0 | 2 | 6 | 1 | 2 | 4 | 15 |
| SP110 | 1 | 2 | 0 | 4 | 3 | 5 | 15 |
