# Supplementary material for: Micro-shear bond strength of 3D printed hybrid ceramic with non-thermal plasma surface treatment: in-vitro study
Source: Sci Rep. 2026 Apr 2;16:11237. doi: 10.1038/s41598-026-43647-w (PMC13046835; doi:10.1038/s41598-026-43647-w)
Supplement: Supplementary file 7 — Supplementary Material 7 [file 41598_2026_43647_MOESM7_ESM.docx]

**Table 1**: Failure mode categorization.

| Types | Description |
| --- | --- |
| Adhesive failure (A) | Interfacial debonding across hybrid ceramic-resin cement adhesive interface |
| Cohesive failure  (C) | C1. Debonding within hybrid ceramic.  C2. Debonding within resin cement. |
| Mixed failure  (M) | M1. Adhesive interface and cohesive hybrid ceramic.  M2. Adhesive interface, cohesive hybrid ceramic, and cohesive resin cement.  M3. Adhesive interface and cohesive resin cement. |
