# Supplementary material for: Micro-shear bond strength of 3D printed hybrid ceramic with non-thermal plasma surface treatment: in-vitro study
Source: Sci Rep. 2026 Apr 2;16:11237. doi: 10.1038/s41598-026-43647-w (PMC13046835; doi:10.1038/s41598-026-43647-w)
Supplement: Supplementary file 8 — Supplementary Material 8 [file 41598_2026_43647_MOESM8_ESM.docx]

**Table 3:** Intergroup comparisons, mean and standard deviation values of micro-shear bond strength (MPa) (**transformed**).

| Groups | Mean ± SD |
| --- | --- |
| PL | 2.35±0.57^AB^ |
| S50 | 2.12±0.75^B^ |
| S110 | 2.18±0.31^B^ |
| SP50 | 2.70±0.49^A^ |
| SP110 | 2.37±0.38^AB^ |
| p-value = 0.026^^[[1]](#footnote-1)^*^ | |
| ^*^ significant (p<0.05). Values with different superscripts within the same **horizontal row** are significantly different and values with same superscripts within the same horizontal raw are insignificantly different. | |

1. [↑](#footnote-ref-1)
