# Supplementary figures and images for: Micro-shear bond strength of 3D printed hybrid ceramic with non-thermal plasma surface treatment: in-vitro study
Source: Sci Rep. 2026 Apr 2;16:11237. doi: 10.1038/s41598-026-43647-w (PMC13046835; doi:10.1038/s41598-026-43647-w)

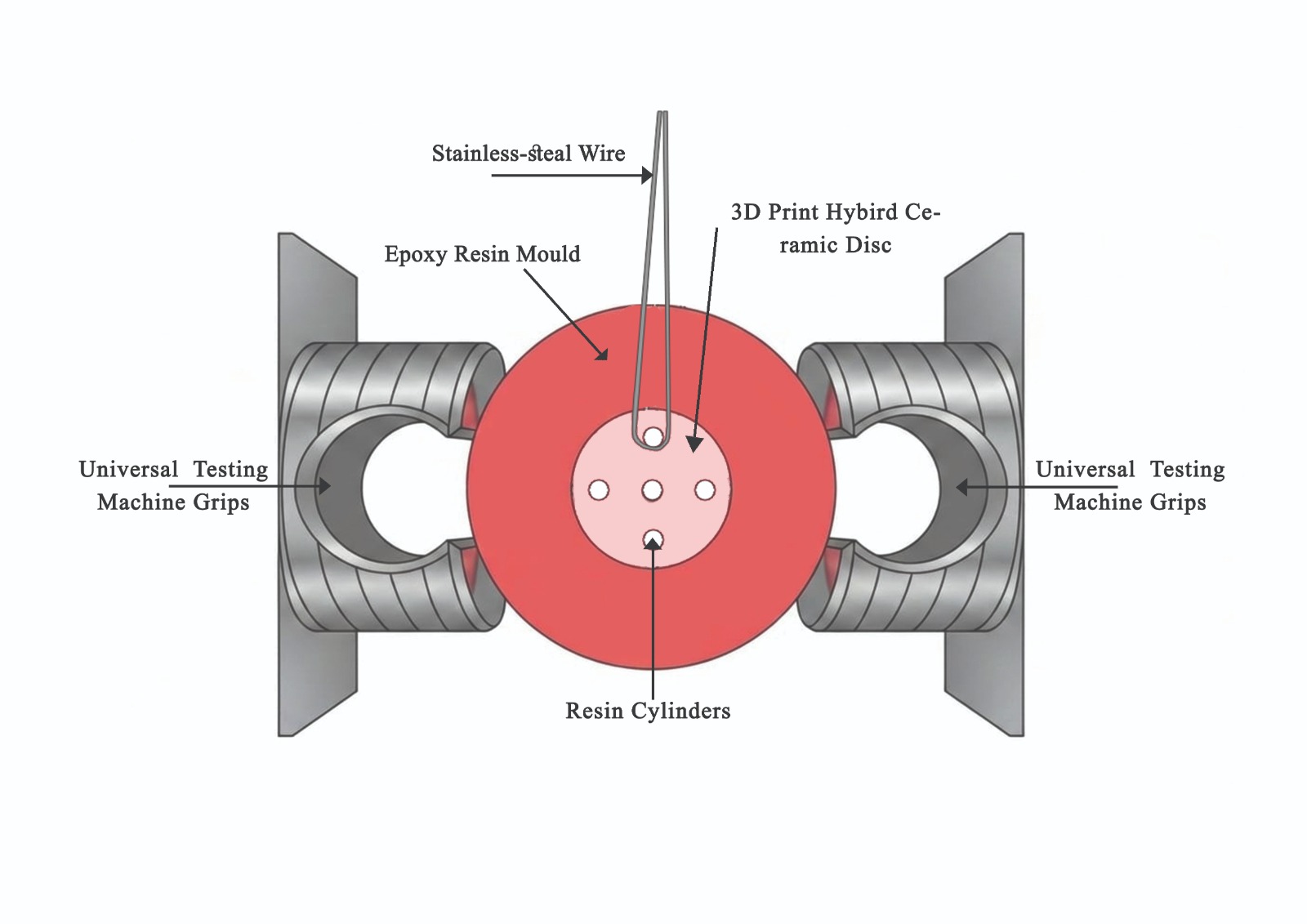

Supplement: Supplementary file 9 — Supplementary Material 9 [file 41598_2026_43647_MOESM9_ESM.jpg]

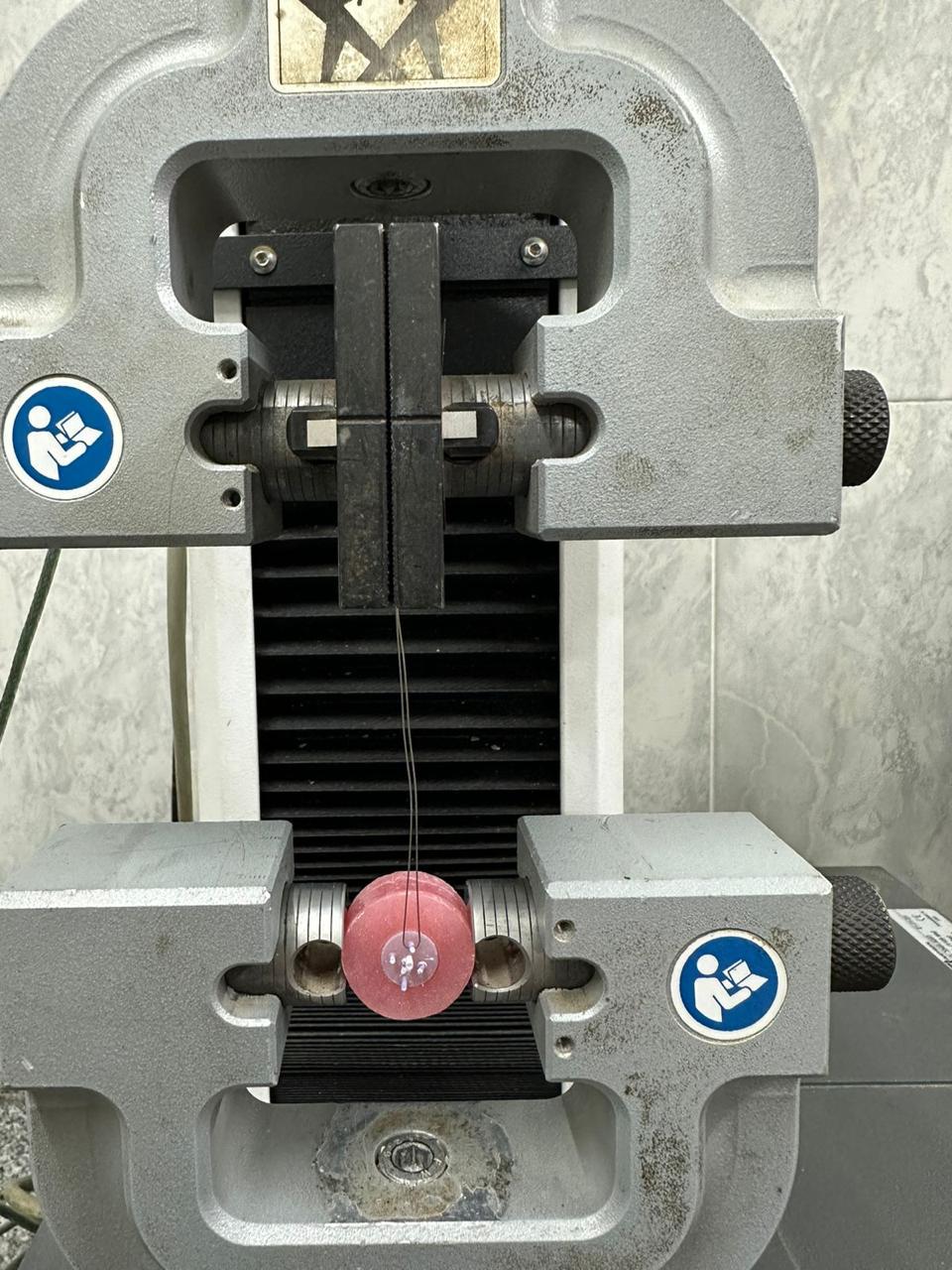

Supplement: Supplementary file 10 — Supplementary Material 10 [file 41598_2026_43647_MOESM10_ESM.jpg]
